# Effects of Carbon Nanotube and Graphene Oxide Incorporation on the Improvements of Magneto-Induced Electrical Sensitivity of Magneto-Rheological Gel

**DOI:** 10.3390/polym14235286

**Published:** 2022-12-03

**Authors:** Daeik Jang, Young-Keun Kim, Taeuk Lim, Hao Cheng, Wonsuk Jung

**Affiliations:** 1 Department of Civil and Environmental Engineering, KAIST, 291 Daehak-ro, Yuseong-gu, Daejoen 34141, Republic of Korea; 2 School of Mechanical and Control Engineering, Handong Global University, 558 Handong-ro, Pohang 37554, Republic of Korea; 3 School of Mechanical Engineering, Chungnam National University, Daejeon 34134, Republic of Korea

**Keywords:** carbon nanotubes (CNTs), carbonyl iron powder (CIP), magneto-resistive, sensors

## Abstract

Magneto-rheological gel (MRG) has been the subject of recent research due to its versatile applications. Especially, the magneto-induced electrical properties of MRGs under different levels of magnetic field enables them to be used as magneto-sensors. However, conventional MRG shows a low level of electrical conductivity, complicating its use in sensor applications. In this regard, in the present study, the carbon nanotube (CNT) and graphene oxide (GO) are added to fabricate new types of MRG. Herein, four different MRG samples were fabricated with reference to an amount of CNT and GO. The microstructural images of carbonyl iron powder (CIP)-based chain structures with CNT and GO were observed using SEM images. Then, their magneto-induced electrical impedances were investigated under four levels of magnetic field (i.e., 0, 50, 100, and 150 mT) and input frequencies (1, 2, 5, and 10 Hz). Based on the experimental results, three electrical models, including first-order series and parallel, and first- and half-order complex models, were proposed, and their accuracy was examined, showing the highest accuracy when first- and half-order complex models were used. The simulated results indicated that the incorporation of both CNT and GO can improve the magneto-induced electrical sensitivity; thus, it can be concluded that MRG with CNT and GO can be a possible method to be used in magneto-sensor applications.

## 1. Introduction

Expeditious developments in research to design a tunable vibration absorber (TVA) are the focus of much recent investigation to satisfy the requirements of various applications for suppressing unwanted vibrations [1,2,3]. Magneto-rheological (MR) materials, including MR elastomers (MRE) and MR gel (MRG), and polymer matrix incorporating carbonyl iron powder (CIP), are regarded as a possible candidates for application in the TVA systems [4,5,6]. The CIP-based chain structures in MR materials are able to be aligned as an external magnetic force is applied to the MR materials, and can change their stiffness and damping, which is called the MR effect [7]. Due to these versatile advantages of MR effects, many studies have proposed the MR materials-based TVA systems [8,9,10]. Jang et al. [8] developed an attachable TVA system using MRE, showing approximately 58% of vibration suppression in the frequency range of 51.6 to 71.9 Hz under 215 mT of magnetic field. Bastola et al. [9] fabricated a TVA based on the MRE, and the fabricated TVA exhibited 730% of stiffness variation when the 520 mT of magnetic field was applied. In addition, Lee et al. [10] developed a MR elastomer-embedded TVA system for a torsional vibration isolation, showing 95.7% of stiffness variation in the frequency range of 16.8 to 23.5 Hz under 150 mT of applied magnetic field.

Recently, many studies have observed the magneto-induced electrical impedances of MR materials under different magnetic forces [11,12,13]. As the magnetic force is applied to the MR materials, the individual CIP can form the CIP-based chain structures, leading to changes in electrical impedance. Wang et al. [11] reported that the electrical impedances of their fabricated MRE changed from 70 kΩ to 20 kΩ as 300 mT of external magnetic field was applied. Zainudin et al. [12] observed that the MRE showed the electrical impedance change from 7.2 kΩ to 3.2 kΩ under 100 mT of external magnetic field. However, according to the previous studies, it was found that the conventional MR materials exhibited high electrical impedances, complicating their use in sensor applications [11,12,13]. To solve such limitations, some researchers have attempted to add carbon-based materials (e.g., carbon nanotube (CNT) and graphite oxide (GO)) with outstanding electrical conductivity into the MR materials to ensure sufficient electrical conductivity for sensor applications [14,15,16,17]; it has been established that the incorporation of CNT or GO can increase the electrical conductivity of the insulated polymeric composites [18,19,20,21]. Many attempts have been made to examine the electrical characteristics of MR materials incorporating carbon-based materials, but fewer investigations have sought a comprehensive study about the effects of carbon-based materials inclusion on magneto-induced electrical impedance change of MR materials [22,23,24] In addition, to the best of the author’s knowledge, studies regarding the comparison of the effects of CNT and GO incorporation on improvements of magneto-induced electrical impedance change of MRG are scarce. 

In this regard, this study aims to investigate the effects of CNT and GO incorporation on the changes of electrical impedance of MRG, and the improvements of electromagnetic sensitivity are systematically examined. Herein, four different MRGs (i.e., pure MRG, MRG with CNT, MRG with GO, and MRG with CNT and GO) were fabricated and their electrical impedances were observed under the four different levels of magnetic field as AC signals with four different frequencies (1, 2, 5, and 10 Hz) were applied to the samples. Based on their magneto-induced electrical impedances, three different electrical models were proposed, and their electromagnetic sensitivities were analyzed.

## 2. Experimental Section

### 2.1. MRG Sample Prepration

**Figure 1:**
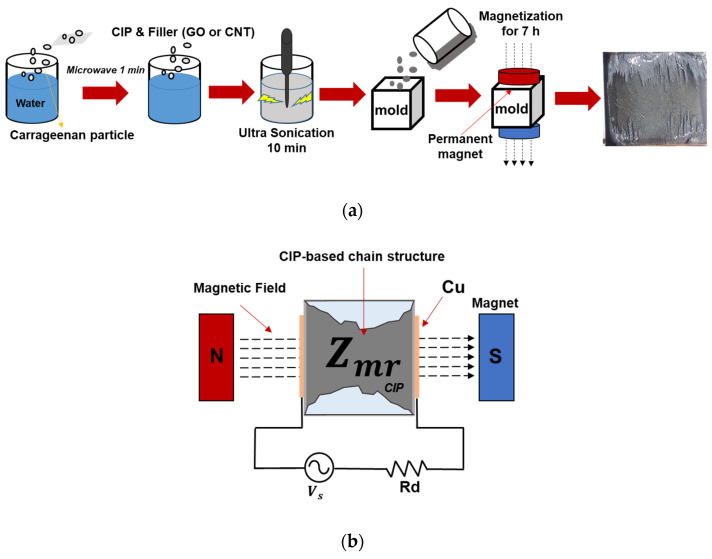
Schematic of (**a**) MRG sample preparation and (**b**) a basic electric circuit for measuring electrical impedance of MRG samples.

The MRG samples were fabricated as shown in Figure 1a. The hydrophilic gelatin base was prepared by adding 0.5 g of carrageenan (No. 34399, MSC CO., Ltd. Yangsanciyt Korea) into 50 g of deionized water. The carrageenan solution was heated using a micro oven for 1 min. Then, the 25 g of CIP (CIP 3189, BASF) with size of 3 μm with conductive fillers including multi-walled CNT (CM-130, Hanwha Chemical) and GO (V50 GO powder, Standard Graphene) were additionally added. The outer diameter, aspect ratio, and density of CNT ranges from 10 to 15 nm, 2 × 10^3^, and 0.05 g/cm^3^, respectively. The particle size of GO is from 37 to 162 μm (D50: 87 μm), and the thickness of GO is about 2 to 2.4 nm. The mixture was thoroughly stirred, and then poured into a square mold with 32 mm width and 30 mm thickness. Finally, the mixture was cooled in a refrigerator for 7 h to completely solidify samples. Four types of MRG samples were prepared considering the amount of CNT and GO. 50% of CIP was added to the all sample, and the details of the mix proportions of the MRG samples are summarized in Table 1.

### 2.2. Experimental Details

Two copper plates were used as electrodes, and they were placed at both sides of the MRG samples. The basic electric circuit, including electrodes, dummy resistor, and function generator, were designed to apply input voltage to MRG samples as seen in Figure 1b. For a dummy resistor, 2 kΩ of resistor was connected in series, which has a value of resistance similar to MRG samples. The electrical impedances of MRG samples were investigated using an oscilloscope (TDS 2022B, Tektronix) under the different levels of magnetic field (0, 50, 100, and 150 mT) as the AC input signal with 1 V and different frequencies (1, 2, 5, and 10 Hz) was applied to the samples. Based on the designed circuit, Formula (1) can be obtained. Then, the electrical impedances of MRG samples can be calculated as Formula (2).

(1) *V_s_* = *V_dummy_* + *V_MR_* = (*R_dummy_* +*Z_MR_*) × *i* = (1 + *Z_MR_*/*R_dummy_*) × *V_dummy_*

(2) *Z_MR_* = *R_dummy_* × (*V_s_*/*V_dummy_* − 1)

## 3. Results and Discussion

### 3.1. Microstructural Images of MRG Samples

**Figure 2:**
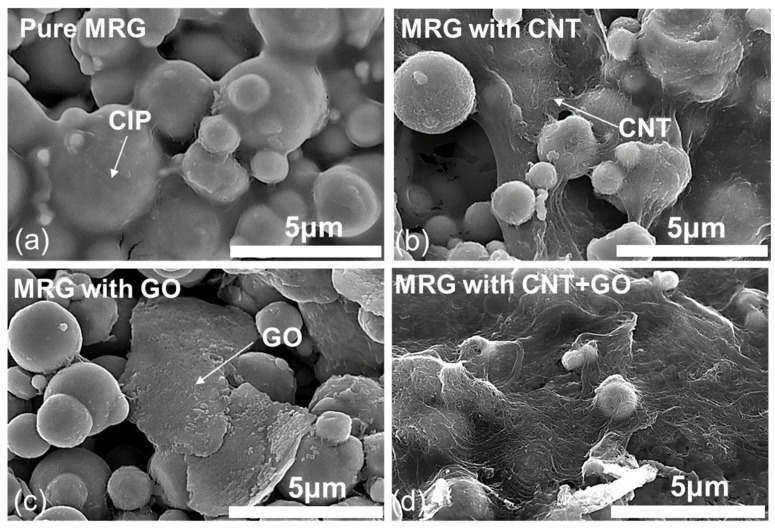
SEM images of (**a**) pure MRG, (**b**) MRG with CNT, (**c**) MRG with GO, and (**d**) MRG with CNT and GO samples.

The CIP-based chain structures with CNT and/or GO are observed using SEM images as shown in Figure 2. As seen in Figure 2, the particle size of CIP is in a broad distribution from 500 nm to 5 μm. Also, it can be found that the length and diameter of CNT and particle size of GO observed in Figure 2 are similar to that summarized above. In addition, the effect of CNT and GO incorporation on the formation of CIP-based chain structure can be found in Figure 2. The individual CNT particles wrapped the CIP and they formed the CIP@CNT clusters. The GO particles were located between the adjacent CIP, acting as a bridge in the CIP-based chain structures. Thus, the incorporated CNT and GO can improve the formation of CIP-based chain structures with enhanced electrical conductivity, which is in close agreement with the previous studies [14].

### 3.2. Magneto-Induced Electrical Impedances of MRG Samples

**Figure 3:**
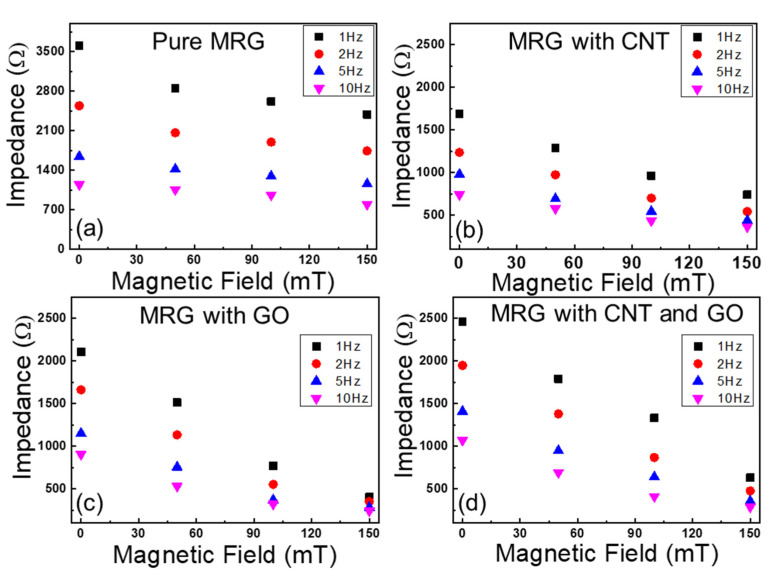
Magneto-induced electrical impedances of (**a**) pure MRG, (**b**) MRG with CNT, (**c**) MRG with GO, and (**d**) MRG with CNT and GO samples.

The electrical impedances of MRG samples under four different levels of magnetic field (i.e., 0, 50, 100, and 150 mT) were measured when AC input voltage with four different frequencies (i.e., 1, 2, 5, and 10 Hz) was applied to the samples, as shown in Figure 3. First, the effect of incorporated conductive fillers on the improvements of electrical impedances can be found. The electrical impedances of samples decreased as conductive fillers (i.e., CNT and GO) were added. As seen in SEM images of Figure 2, the added CNT and GO can improve the possibility of forming CIP-based conductive networks, leading to the decrease in electrical impedance. Second, the effect of input frequencies on changes of electrical impedances of the samples can be observed. As seen in Figure 3, the electrical impedance of all samples decreased as the input frequencies increased. This can be explained from the properties of capacitance which are inversely proportional to the input frequency, and this phenomena can be found in the previous studies [25]. Third, it can be established that the electrical impedances of MRG samples regardless of the added conductive fillers decreased as the applied magnetic field increased. As the magnetic field is applied to the samples, CIP with and without conductive fillers can be aligned in chain-structures; thus, it can help electrons to move easily through the conductive networks.

Based on the electrical impedances values observed in Figure 3, the fractional change in resistances (FCR) can be examined as the different levels of magnetic field are applied to the MRG samples. The fractional change in resistance can be calculated as following Equation (3).

**Figure 4:**
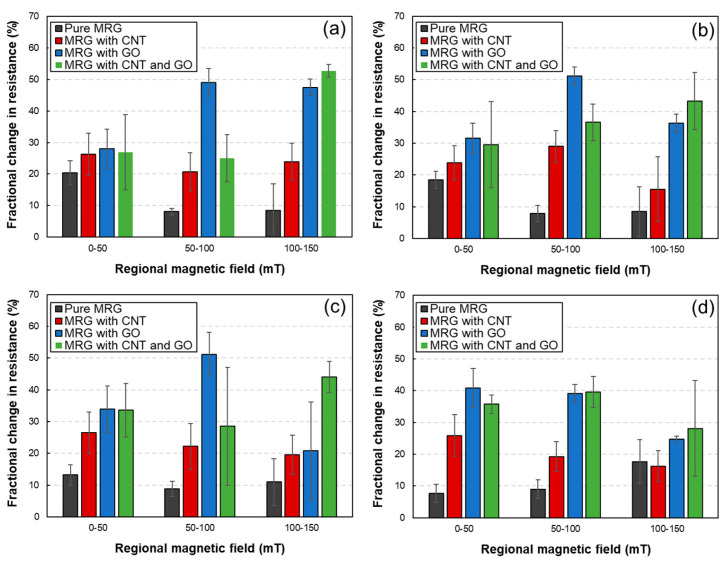
Fractional change in resistance of MRG samples under the different input frequencies (**a**) 1 Hz, (**b**) 2 Hz, (**c**) 5 Hz, and (**d**) 10 Hz.

(3) FCR (%) = Abs{(Zmag − Z_0_)/Z_0_ × 100} (%)

where Z_mag_ and Z_0_ denote the electrical impedances of MRG with and without applying magnetic field, respectively. This FCR value implies the magneto-induced sensitivity; thus, these values are used to compare the sensitivity of each sample. In Figure 4, it can be found that the magneto-induced sensitivity increased as the levels of applied magnetic field increased. This phenomenon can be inferred from the formation of CIP-based chain structures. The well-aligned chain structure can be formed as the magnetic field increases, leading to the high values in electrical impedance changes. In addition, it can be observed that the MRG samples incorporating conductive fillers (i.e., MRG with CNT, MRG with GO, and MRG with CNT and GO) showed improved magneto-induced sensitivity compared to that of pure MRG sample. As found in the previous results, the additionally incorporated fillers (CNT and GO) can help to form the CIP-based conductive networks. Thus, the improved conductive networks are more sensitive under the magnetic field, increasing FCR values. However, interestingly, the FCR values decreased as the CNT is added. This can be deduced from the shape of the conductive fillers, indicating the fiber-typed CNT and plate-typed GO. When the CNT is incorporated to the MRG, the fiber-typed CNT can easily form conductive pathways. Therefore, even though the CIP can change its original position when the magnetic field is applied, the well-formed conductive networks consisting of CNT are already developed. In contrast, the plate-typed GO can easily detach from the adjacent GO compared to that of fiber-typed CNT. For these reasons, it can be deduced that magneto-induced electrical sensitivity decreases when the fiber-typed CNT is added to the MRG.

### 3.3. Electrical Impedance Prediction Using Electrical Circuit Models

**Figure 5:**
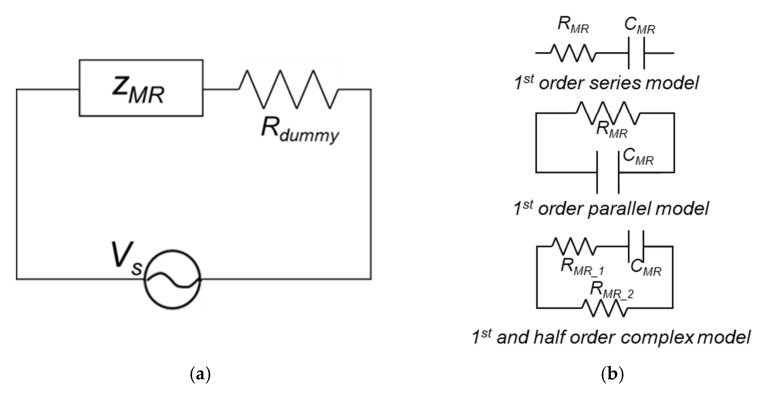
Schematic of (**a**) basic circuit used in modeling and (**b**) three different electrical impedances model of MRG samples.

Based on the experimental results, the electrical impedances models were used to design the basic circuit, as illustrated in Figure 5. Herein, three different models including first order series, first order parallel, and first—half order complex models were proposed to express the electrical impedances of the MRG samples. The electrical impedances of MRG samples using three different models can be calculated as follows, according to the previous study [26].

*Z_MR_series_* = *R_MR_* + (*jwC_MR_*)^−1^ (4)

*Z_MR_parallel_* = *R_MR_* +(1 + *jwR_MR_C_MR_*)^−1^ (5)

*Z_MR_complex_* = *R_MR_*__2_ + (*R_MR_*__2_ + *R_MR_*__1_*R_MR_*__2_*C_MR_*) × ( 1 + *jwR_MR_*__1_*C_MR_*)^−1^ (6)

**Figure 6:**
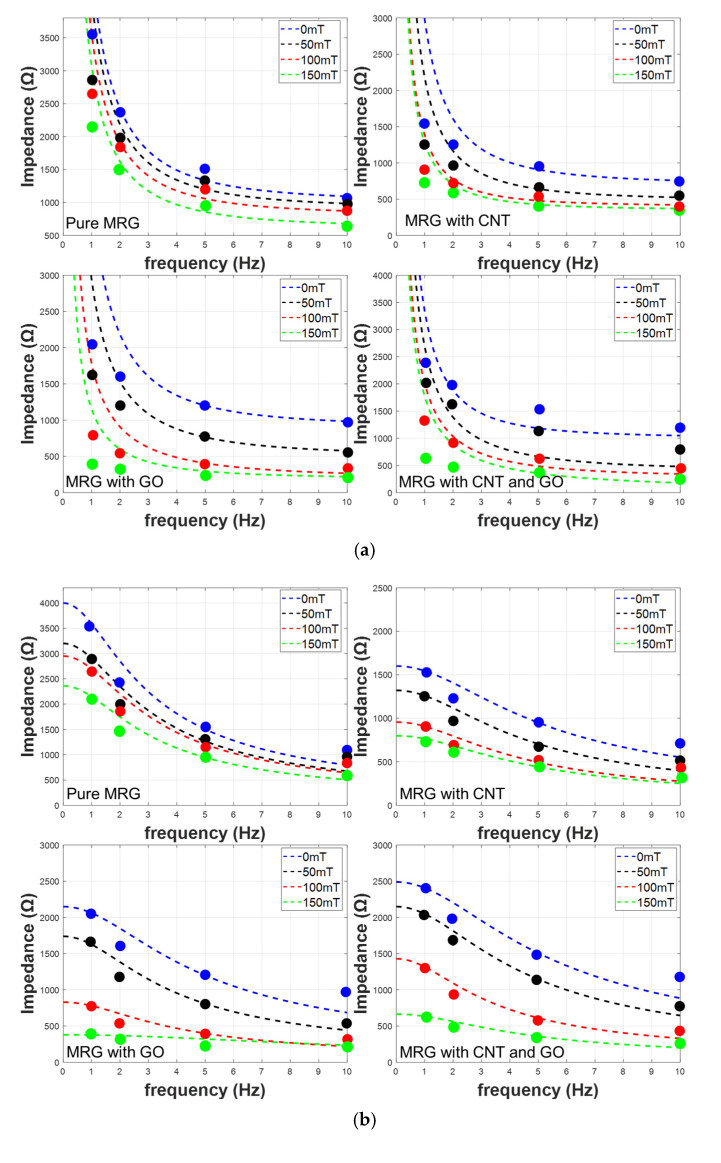

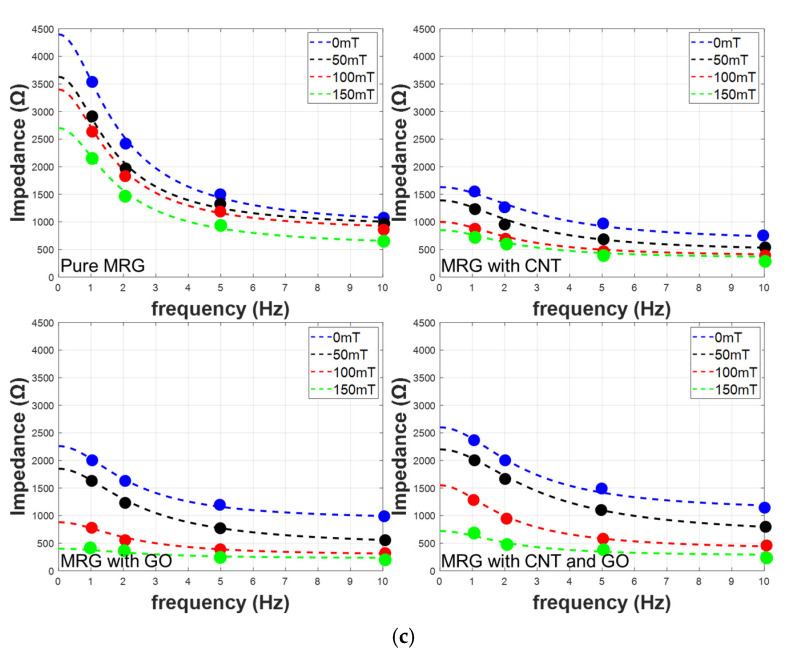
Variation in electrical impedance of MRG samples and curve fitted results using (**a**) first order series, (**b**) first order parallel, and (**c**) first- and half-order complex models.

According to Equations (4) to (6), the relationship between electrical impedances and input frequency under the different magnetic fields can be obtained, as shown in Figure 6. As seen in Equations (4) to (6), the electrical impedances are inversely proportional to the frequency; thus, the decreasing of electrical impedances can be seen as increasing in frequency (see Figure 6). Interestingly, the effects of the applied magnetic field on the changes of electrical impedances decrease when the input frequency is increased. This can be deduced from Equations (4) to (6), indicating the combination of resistance and capacitance. For a low frequency, the magnetic field can affect the values of resistances and capacitance. However, as the input frequency increases, the effects of terms consisting of capacitance decrease; thus, the electrical impedances are mostly affected by the magneto-induced electrical resistances. Therefore, the degree of magneto-induced electrical impedances are decreasing as the input frequency increases.

**Figure 7:**
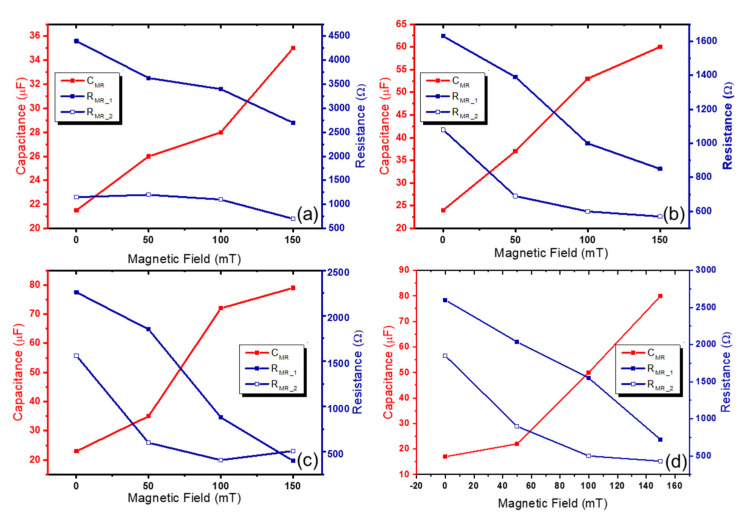
Simulated values of resistance and capacitance under the different magnetic field using first- and half-order complex models of (**a**) pure MRG (**b**) MRG with CNT, (**c**) MRG with GO, and (**d**) MRG with CNT and GO samples.

In addition, in Figure 6, the different accuracy can be found when three different models are used, and the measured R-squared values are shown in Table 2. Among the three models, the electrical impedances calculated using the first- and half-order complex model showed R-squared values higher than 0.97 under all levels of magnetic field. These values are much higher than those found with other models. Thus, the variation of electrical parameters including *R_MR_*__1_, *R_MR_*__1_, and *C_MR_* was observed using the first- and half-order complex model. In Figure 7, the relationship between electrical parameters and magnetic field is exhibited, and the degree of changes in electrical parameters are summarized in Table 3. It can be seen that the incorporated conductive fillers (i.e., CNT and GO) can improve the magneto-induced electrical sensitivity. For a pure MRG sample, the increasing and decreasing of capacitance and resistance was about 63% and 39%, respectively. These values increased to 150% and 47% in a MRG with CNT sample, 240% and 82% in a MRG with GO sample, and 370% and 72% in a MRG with CNT and GO sample, respectively. The improvement of magneto-induced electrical sensitivity can be deduced from the well-formed conductive networks, and the degree of movement of CIP-based chain structure. As the conductive fillers are added, the possibility of contact area with respect to the fillers and CIP increase, leading to the higher changes in electrical properties. In this regard, the authors can conclude that the incorporating of conductive fillers can improve the magneto-induced electrical sensitivity. Especially, the synergistic effects of CNT and GO on increasing electrical sensitivity can be found, and incorporating both CNT and GO into MRG can be a possible method to use MRG within magneto-sensor applications.

## 4. Conclusions

In the present study, the effects of CNT and GO incorporation on the improvements of magneto-induced electrical sensitivity of MRG were systematically investigated. First, four different MRG samples regarding the types of added fillers (i.e., pure MRG, MRG with CNT, MRG with GO, and MRG with CNT and GO) were fabricated. The CIP-based chain structures with CNT and GO were observed with SEM images. Then, their magneto-induced electrical sensitivity under four different levels of magnetic field (0, 50, 100, and 150 mT) and input frequencies (1, 2, 5, and 10 Hz) were examined. Based on the experimental results, the three different electrical models (first order series and parallel, and first- and half-order complex models) were proposed to predict their magneto-induced electrical sensitivity. The R-squared values are adopted to observe the accuracy of the proposed models. The simulated results showed that the first- and half-order complex model can exhibit the higher accuracy (>0.97). In addition, the incorporation of both CNT and GO can lead to 72% and 370% in decreasing resistance and increasing capacitance, respectively; while 39% and 63% were exhibited in MRG without any fillers. In future works, the scale effects on magneto-induced electrical sensitivity of CNT and GO-embedded MRG under the high levels of magnetic field will be carried out to apply the fabricated samples for practical and commercial applications. In addition, the rheological characterization and mechanical characteristics of the proposed MRG will be investigated.

## Figures and Tables

**Table 1:** Mix proportions of the MRG samples in the present study (g).

Sample	Water	Carrageenan	CIP	CNT	GO
Pure MRG	100	1	50	0	0
MRG with CNT	100	1	50	0.5	0
MRG with GO	100	1	50	0	0.5
MRG with CNT and GO	100	1	50	0.25	0.25

**Table 2:** R-squared values of MRG using different prediction models (**a**) first order series (**b**) first order parallel, and (**c**) first- and half-order complex models.

**Sample**	**(a)**			
	**0 mT**	**50 mT**	**100 mT**	**150 mT**
Pure MRG	0.9676	0.9769	0.9673	0.9571
MRG with CNT	0.9096	0.9409	0.9447	0.9289
MRG with GO	0.9447	0.9535	0.9897	0.9395
MRG with CNT and GO	0.8966	0.9054	0.9758	0.9818
**Sample**	**(b)**			
	**0 mT**	**50 mT**	**100 mT**	**150 mT**
Pure MRG	0.9619	0.9405	0.9515	0.9664
MRG with CNT	0.9477	0.9400	0.9281	0.9349
MRG with GO	0.9275	0.9567	0.9004	0.8067
MRG with CNT and GO	0.9451	0.9768	0.9487	0.9131
**Sample**	**(c)**			
	**0 mT**	**50 mT**	**100 mT**	**150 mT**
Pure MRG	0.9937	0.9914	0.9948	0.9938
MRG with CNT	0.9865	0.9869	0.9920	0.9983
MRG with GO	0.9883	0.9896	0.9748	0.9949
MRG with CNT and GO	0.9882	0.9949	0.9916	0.9941

**Table 3:** Electrical parameters of changes of MRG samples using first- and half-order complex model.

Sample	Increasing of Capacitance	Decreasing of Resistance
Pure MRG	63%	39%
MRG with CNT	150%	47%
MRG with GO	240%	82%
MRG with CNT and GO	370%	72%

## Data Availability

The data presented in this study are available on request from the corresponding author.
